# The Late Fire in Boston

**Published:** 1872-11

**Authors:** 


### THE LATE FIRE IN BOSTON.

As our readers know a most destructive fire has swept away a large portion of the business portion of the city of Boston. We sincerely hope our cotemporaries of that city—The Boston Medical and Surgical Journal, The Journal of the Gynæcological Society, The Journal of Chemistry, and Good Health Journal have escaped.

We are glad to learn that Messrs. Codman & Shurtleff, who are advertisers in our Journal, have escaped. While we and they sympathize with those who were not so fortunate, we rejoice with our friends above that they came through without loss. They therefore, assure our readers of their facilities for meeting their wants, and the desire to do so to their entire satisfaction, remains undiminished.

